# A new Achilles Heel in breast cancer?

**DOI:** 10.18632/oncotarget.1178

**Published:** 2013-07-15

**Authors:** George Sflomos, Cathrin Brisken

**Affiliations:** Swiss Institute for Experimental Cancer Research, National Center of Competence in Research Molecular Oncology, School of Life Sciences, Ecole Polytechnique Fédérale de Lausanne, CH-1015 Lausanne, Switzerland; Swiss Institute for Experimental Cancer Research, National Center of Competence in Research Molecular Oncology, School of Life Sciences, Ecole Polytechnique Fédérale de Lausanne, CH-1015 Lausanne, Switzerland

**Figure 1:**
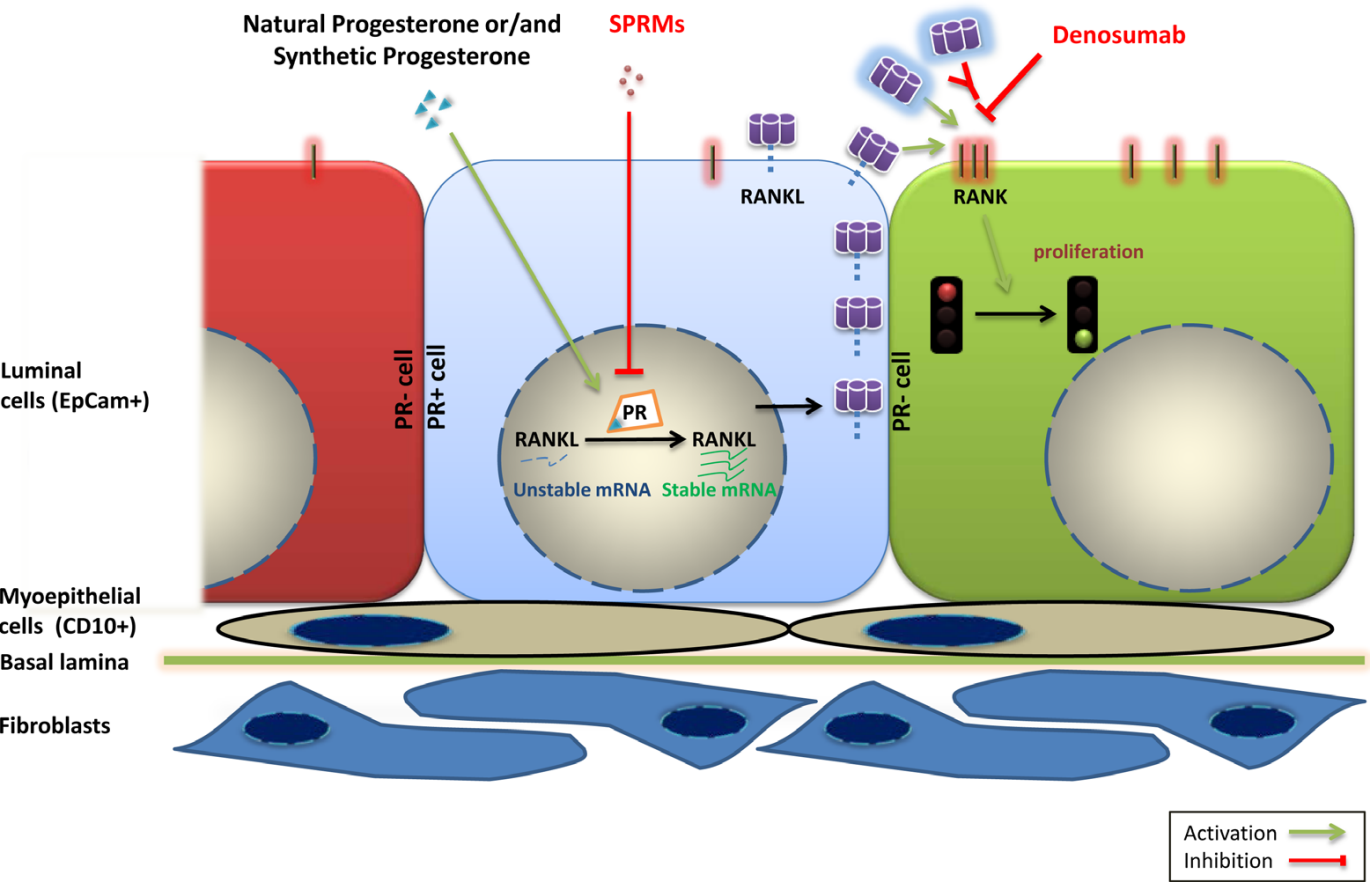
Model of the PR/RANKL signaling axis in the human breast Activation of PR signaling by natural progesterone or progestins leads to increase in RANKL protein levels in PR positive luminal breast epithelial cells (central cell in light blue) mainly through stabilization of mRNA. RANKL protein binds to RANK receptor (depicted on the adjacent green cell to the right) and activates this pathway leading to cell proliferation. SPRMs and drugs that target RANKL pathway such as denosumab may hence inhibit breast cell proliferation. (PR: Progesterone Receptor, SPRM: Selective Progesterone Receptor Modulators, CD10: Cluster of Differentiation 10, EpCam: Epithelial Cell adhesion molecule, RANK: Receptor activator of nuclear factor kappaB, RANKL: Receptor activator of nuclear factor kappaB ligand).

The female sex hormones, estrogens (E) and progesterone (P) are major determinants of postnatal mammary gland development and are thought to promote breast carcinogenesis. How do they impact on the human breast? The most widely used models to study E and P signaling are hormone receptor positive breast cancer cell lines. However, it is unclear to what extent they provide insights into the breast tissue molecular pathophysiology. The study (*Tamara T., Sflomos G et al., Science Translational Medicine, 2013, Vol. 5, Issue 182, p. 182*) presents and validates a novel *ex vivo* culture model based on tissue microstructures from fresh human reduction mammoplasty specimens. For the first time, physiological hormone action can be studied in a human model and new opportunities to unravel hormone action open up. The work revealed Progesterone/RANKL axis as a major driver of cell proliferation in the adult human breast with potential clinical implications for breast cancer patients. Current knowledge about the mechanisms by which female reproductive hormones control mammary gland development and breast carcinogenesis stem mainly from animal studies. In particular, the mouse model, in which genetic tools can be combined with powerful tissue recombination techniques, has been instrumental in revealing that epithelial estrogen receptor (ERα) signaling controls pubertal gland development whereas progesterone receptor (PR) signaling is the major proliferative stimulus in the adult mouse mammary gland. Across species, 30-50% of the breast epithelial cells express the ERα and PR. The mouse model revealed that at the cellular level, hormone action relies largely on paracrine signaling. In particular, receptor activator of nuclear factor kappaB ligand (RANKL) is a central mediator of PR-induced cell proliferation. RANKL, a transmembrane type II protein is a member of the tumor necrosis factor superfamily and signals through its receptor RANK (Figure 1). Upon RANKL binding, RANK receptor elicits activation of a signaling cascade important for mammary gland development and tumorigenesis.

Epidemiological studies have linked menstrual cycles to breast cancer risk and it is conceivable that the associated peaks of serum progesterone levels could lead to RANKL induction and subsequent cell proliferation. Such a scenario has clinical implications because drugs that target PR/RANKL have been developed to treat reproductive disorders and bone diseases, respectively. If the same pathway is involved in human breast carcinogenesis, these drugs may be used for breast cancer prevention in high-risk patients who are premenopausal to interfere with tumor promoting effects of menstrual cycles.

When primary human breast epithelial cells (HBECs) are grown on tissue culture plastic they lose hormone receptor expression and cannot be used to study hormone action. Hormone receptor positive breast cancer cell lines such as MCF-7 and T47D express high levels of hormone receptors *in vitro* and are widely used models. However, estrogen and progesterone targets identified in these cell lines differ largely from those identified in the mouse mammary gland *in vivo.* Whether these discrepancies are due to differences between mice and humans, relate to differences between *in vitro* and *in vivo* assays and/or result from changes that occurred during oncogenic transformation is unclear.

In light of the importance of paracrine signaling in the mouse mammary gland we argued that it is important to maintain intercellular interactions. We minimally treated fresh human breast reduction mammoplasty samples to obtain tissue microstructures. In this *ex vivo* approach, breast cells retain hormone receptor expression and remain hormone responsive. Interestingly, estrogens failed to elicit cell proliferation in a consistent fashion whereas progesterone signaling induced proliferation in most samples. Activation of progesterone receptor signaling did not affect RANKL expression in MCF-7 and T47D cell lines but induced it in the tissue microstructures. RANKL induction by progesterone receptor signaling is mediated mainly by molecular mechanisms controlling RANKL's mRNA maturation and stability, leading to elevated amount of this protein in the cell (Figure 1).

An important question that arises from these observations is whether the PR/RANKL axis that operates in the normal breast epithelium is also active in primary breast cancers? If RANKL continues to drive cell proliferation in breast carcinomas, cancer patients might benefit from drugs that inhibit RANKL. Denosumab is a humanized antibody that blocks RANKL signaling. It has been FDA approved for the treatment of bone disorders and is generally well tolerated. As such it could quickly be tested and given to breast cancer patients. While a role, at least during early stages of carcinogenesis is well established in the mouse model, such a role of RANKL in human breast cancer is less clear. This may in part be attributable to the lack of good antibodies to study protein expression in tumor samples.

Anti-estrogens are mainstay of breast cancer treatment both for advanced-stage disease and for the adjuvant settings. The new findings of this study suggest that it is worth reconsidering drugs that target progesterone receptor signaling. New generation selective progesterone receptor modulators (SPRMs) have been developed for gynecological disorders and have fewer side effects compared to previous generations. If the paracrine axis persists in patients bearing PR+ breast cancers then these patients may benefit from these drugs.

Large studies of women on hormone replacement therapy have revealed puzzling results. The intake of combined estrogen and progestin preparations increased breast cancer risk whereas therapy with estrogen and natural progesterone did not. The *ex vivo* system can be useful to address whether progesterone and progestins differ in their ability to induce RANKL and/or other targets and promote cell proliferation not only from microstructures derived from reduction mammoplasties but also from tumor samples.

Over decades, the approval of oral contraceptives has substantially improved birth control and prevented unwanted pregnancies. However, many studies argue that contraceptive pills, most of which include progestins, can increase breast cancer incidence for reasons that are not yet fully understood. Additional bench work using the human microstructure model will help us to identify the molecular underpinnings of these differences and to determine individual risk factors related to new formulations of contraceptives and to a woman's age or reproductive status. This new experimental model holds promise to help us gain newinsights into hormone action in the human breast that is ever so important to tumor development in this organ.

